# Manipulating Foil

**Published:** 1869-05

**Authors:** H. Scott


### MANIPULATING FOIL.

BY H. SCOTT.

Unquestionably we mutually all gain by communicating specificately our various methods of doing things, and I shall hold that it is incumbent on every Dentist to "tell his experience," for such a course can not result otherwise than each one receiving more, in the aggregate, than he contributes. And yet we often become confused in reading this, or that man's way of working, because it is foreign to our experiences. I have seen statements of modes of operating that I thought I could not master to save my life, and believed that no one else could. Still, we measure men's abilities in a matter we have some understanding of, by the way they talk and write, and we learn thereby. We all aim at excellence, I suppose, or, at least, we should do so in our profession or our specialties, and undoubtedly the road to it is untiring diligence and free interchange of thought and experience. General principles are of course the same.

I have failed utterly in attempting to use gold foil in the way that some have said had proved highly successful in their hands. Others may fail, perhaps, in attempting to follow my method. But the way I manage my foil is satisfactory to me, and I can make better fillings by my method than I can by any others that I have tried. I am not afraid of spoiling my foil by touching it with my hands. On the contrary, I manipulate entirely with my fingers; but, in doing so, I keep them comparatively dry by drawing them briskly over my gown, or some part of my garments, every few seconds while handling the foil, which removes the secretions as fast as they come upon the surface. My pellets, balls and wedges being prepared, and my cavity dried out with bibulous paper, (blue) I pass them through my spirit flame successively as I carry them to the cavity. In doing this I use plugging forceps, and if my fingers have been kept dry, as above, there is no appearance of smoke from the pellets as they pass out of the flame. I find my gold thus managed adhering as the pellets successively touch each other, and that it mallets down into a solid mass; at least, as much so as gold foil ever does under any circumstances, and will take as fine a finish as one chooses to put upon it. This is my present method of using gold foil, after having tried in former years various others. Those who are pleasing themselves with the quality of their fillings conducted by different means, should not abandon them for mine, though I suggest that any who may not be satisfied with the method they have been trying, should use the foil as I do, and then determine upon its merits. I use mostly No. 6 soft foils. I use blue tissue paper prepared, for drying, because its color determines when the cavity is thoroughly dry.

I have other experiences I wish to speak of. Where I find great difficulty in controlling the saliva, when about to fill a lower tooth, even with my practice once before given in the REGISTER, (March, 1868) I disregard the whole thing and fill the cavity "submarine." In these cases I manipulate the foil pretty solidly with the fingers, in as large balls and wedges as I can introduce, and then mallet them home as firmly as gold will admit of, always having my cavities of the dove-tail shape, and putting in the last wedge into a corner that is prepared also of the dove-tail shape. Such work, though done under water, if well done will no more give way than a good brick arch can fall. And further, I can cut away the extra gold and finish as perfectly as work done in the dry. I don't say the gold adheres, but I do say that such fillings can be made to answer all the requirements, and we want nothing more. I made such fillings more than twenty years ago, that are as good now as work done in the dry at the same time. Indeed, most of them are entirely perfect.

